# The presence of six potentially pathogenic viruses in pigs suffering from post-weaning multisystemic wasting syndrome

**DOI:** 10.1186/s12917-014-0221-8

**Published:** 2014-09-30

**Authors:** Michaela Vlasakova, Valeria Leskova, Ivan Sliz, Anna Jackova, Stefan Vilcek

**Affiliations:** Department of Epizootiology and Parasitology, University of Veterinary Medicine and Pharmacy, Komenskeho 73, Kosice, Slovakia

**Keywords:** Post-weaning multisystemic wasting syndrome (PMWS), Multiple infections, Vaccination, Porcine circovirus type 2 (PCV2), Porcine reproductive and respiratory syndrome virus (PRRSV), Torque teno sus virus (TTSuV), Porcine teschovirus (PTV), Porcine bocavirus 1 (PBoV1)

## Abstract

**Background:**

Porcine circovirus type 2 (PCV2) is an etiological agent of porcine circovirus diseases (PCVDs). Post-weaning multisystemic wasting syndrome (PMWS) as the most important PCVD is considered a multifactorial disease. It was demonstrated that not only PCV2 but several viruses are associated with PMWS. Studies of viral co-infections in PMWS pigs led often to controversial results. The aim of this work was to determine the presence of emerging (PRRSV), re-emerging (PTV) and newly-emerging (TTSuV1, TTSuV2, PBoV1) viruses in samples of dead pigs suffering from PMWS. The impact of vaccination against PCV2 and the influence of age on the occurrence of single and multiple viral infections in pigs were also investigated.

**Results:**

Viruses were detected by PCR, RT-PCR and real-time PCR in the pooled tissue samples (lymph nodes, liver and spleen) of pigs with PMWS (n = 56) which were divided into three groups: suckling piglets, post-weaning pigs and fattening pigs. In addition, lymph node samples were collected from apparently healthy fattening pigs (n = 59). The effect of vaccination against PCV2 with Ingelvac CircoFlex vaccine was also investigated. Between non-vaccinated pigs, the highest prevalence of individual viruses and multiple viral infections were found in diseased post-weaning and fattening animals with PMWS. Severe clinical disease was observed in swine co-infected with PCV2 and PRRSV. The prevalence of TTSuV1 and TTSuV2 was high in all groups of pigs and did not appear to have a significant effect on the syndrome. Simultaneous infection with TTSuV1 and PBoV1 was frequently confirmed in pigs with PMWS. No healthy pig was found to be infected with PRRSV, PTV or PBoV1. Vaccination against PCV2 did not influence the prevalence of TTSuVs, but significantly protected pigs against multiple viral infections.

**Conclusions:**

Post-weaning PMWS pigs were more often co-infected with viral pathogens than suckling or fattening pigs. Co-infection with PRRSV enforces clinical signs of PMWS, the influence of other viral co-infections is not clear. Vaccination against PCV2 significantly reduced viral co-infections in pigs.

## Background

Porcine circovirus type 2 (PCV2) as an etiological agent of porcine circovirus diseases (PCVDs) was firstly identified in the 1990s in Canada [1], and was later detected and characterized in USA and Europe [2,3]. In the Czech Republic and Slovakia PCV2 in pigs was confirmed and genetically typed a few years later [4–6].

Over the last five years, due to the world financial depression, weak government support of farmers and infectious diseases, the number of pigs in Slovakia has decreased dramatically while numbers in the Czech Republic have remained stable. At the end of 2013, almost 519 000 pigs, including up to 39 000 sows were registered in Slovakia, but little pig breeding is done and all-in/all-out swine fattening systems predominate. Beside the national husbandry, a few farms are managed by Danish companies. In the Czech Republic, approximately 1.5 million of pigs are housed, including 100 000 sows. Similar to Slovakia, all-in/all-out systems are dominant. Pigs are mostly imported from Denmark and Netherlands to both countries.

Post-weaning multisystemic wasting syndrome (PMWS) as the most important PCVD is considered a multifactorial disease [7]. In the syndrome development, several viruses and bacteria are involved [8,9]. Several scientific papers indicate that an increase of post-weaning mortality with significant impact on pig production worldwide is caused by the circulation of several viral pathogens on farms [8,10,11]. During PMWS outbreaks PRRSV is frequently present in pig herds [12,13]. It was considered that also Torque teno sus virus 1 (TTSuV1) and Torque teno sus virus 2 (TTSuV2) play a role in diseases associated with PCV2, but controversial results were revealed [14–16]. The prevalence of Porcine bocavirus 1 (PBoV1) in swine populations has been studied recently to see if the virus has any effect on PMWS development [16].

The aim of this study was to determine the presence of emerging (PRRSV), re-emerging (Porcine teschovirus - PTV) and newly-emerging (TTSuV1, TTSuV2, PBoV1) viruses in pigs at the terminal stage of PMWS. The occurrence of single and multiple viral infections in pigs of different age categories was also investigated. The impact of vaccination against PCV2 was determined on the limited level on healthy fattening animals.

## Methods

None of the pigs were killed with the purpose to fulfill the objectives of the present study. All diseased animals were naturally infected and their tissue samples were collected after the death as the consequence of the pathological process. Samples acquisition was performed with the agreement of the pig farmers. Pooled tissue samples (lymph nodes, liver and spleen) from domestic pigs originating from 10 farms in Slovakia and 12 farms in the Czech Republic were collected during a period of six years between 2007 and 2012. These samples were originally sent to diagnostic laboratories of The State Veterinary Institutes for the confirmation of diagnosis. The samples were then passed to our laboratory for further investigation. Specimens from the healthy animals were obtained on the abattoir during official meat inspection. Sampling of these specimens adhered to the Council Regulation (EC) No. 1099/2009 and the Statutory Order of the Slovak Republic No. 432/2012 on the protection of animals at the time of killing.

A collection of 115 tissue samples included in this study originated from diseased (n = 56) and healthy (n = 59) animals.

Dead diseased pigs were divided into three groups: suckling piglets – between 4 and 6 weeks of age (n = 11), post-weaning pigs – between 7 and 10 weeks of age (n = 14) and fattening pigs – between 11 and 24 weeks of age (n = 31). Clinical signs of PMWS (diarrhea, inappetence, wasting and enlargement of lymph nodes) were recorded in all 56 diseased pigs by veterinarian at the time of sampling. Nineteen of these animals also suffered from unspecified respiratory tract disorders. To confirm PMWS, the generally accepted criteria for diagnosis of PMWS were applied: clinical manifestation, characteristic histopathological lesions in lymphoid tissues and detection of PCV2 within the tissues [17]. The application of this complex diagnostic approach led to the confirmation of PMWS in 33 pigs. In the remaining 23 animals histopathological examination of lymphoid tissues was not performed but because the other criteria were fulfilled, these pigs were labeled as PWMS-suspected. PMWS (n = 33) and PMWS-suspected (n = 23) pigs formed a group of diseased pigs. None of the diseased animals had been vaccinated against PCV2.

Lymph node samples were collected from additional fifty nine apparently healthy 22 to 28 week old fattening pigs following slaughter in an abattoir. All healthy animals originated from two Slovak swine farms. Vaccination of piglets at the age of 3 weeks against PCV2 with Ingelvac CircoFlex vaccine (Boehringer Ingelheim, Germany) was carried out on the first farm (n = 30). Non-vaccinated animals were housed on the second farm (n = 29). Tissue samples from younger healthy pigs were not available for ethical reasons, since it would have demanded killing of healthy animals.

Clinical samples were prepared as 20% w/v tissue homogenates in PBS (Calbiochem, Germany) or nuclease-free water (SERVA Electrophoresis, Germany). Total DNA (PCV2, TTSuV1, TTSuV2 and PBoV1) and RNA (PRRSV and PTV) were extracted from 300 μl or 200 μl of tissue homogenates using Chelex resins (Bio-Rad, USA) and TRIzol reagent (Ambion, USA), respectively, according to manufacturer’s recommendations. For detection of RNA viruses, reverse transcription using gene-specific primer (for PRRSV) or random hexamers (for PTV) followed by the PCR was performed. DNA viruses were analyzed using PCR or real-time PCR based on SYBR Green. The references concerning the detection of specific PCR products are listed in Table 1.

**Table 1:** **PCR primers used for amplification of PCV2, PRRSV, TTSuV1, TTSuV2, PTV and PBoV1**

**Virus**	**Primers (5’ → 3’)**	**Lenght of amplification**	**Genome region amplified**	**References**
**PCV2**	F: TAGGTTAGGGCTGTGGCCTT	263 bp	ORF2	LaRochelle et al. [18]
	R: CCGCACCTTCGGATATACTG			
**PRRSV**	F: GCCCCTGCCCAICACG	637 bp 505 bp	ORF7	Oleksiewicz et al. [19] Drew et al. [20]
	R: TCGCCCTAATTGAATAGGTGA			
	F: GTGCTGGGCGGCAAACGAGCTGGT			
	R: TCGCCCTAATTGAATAGGTGACTC			
**TTSuV1**	F: CGGGTTCAGGAGGCTCAAT	305 bp	UTR	Segalés et al. [21]
	R: GCCATTCGGAACTGCACTTACT’			
**TTSuV2**	F: TCATGACAGGGTTCACCGGA	252 bp	UTR	Segalés et al. [21]
	R: CGTCTGCGCACTTACTTATATACTCTA			
**PTV**	F: AGTTTTGGATTATCTTGTGCCC		5’-UTR	Zell et al. [22] Krumbholz et al. [23]
	R: CGCGACCCTGTCAGGCAGCAC	316 bp 158 bp		
	F: TGAAAGACCTGCTCTGGCGCGAG			
	R: GCTGGTGGGCCCCAGAGAAATCTC			
**PBoV1**	F: GCATTGCAAGAAGCTGAAGC	992 bp 680 bp	VP1/VP2	this study
	F: CATTGAAGAGGTTGAAGTGGA			
	R: TTTCTTCTCCTGTTCTTAGTA			

PCR products were analyzed in 2% agarose gel and visualized under UV light after GelRed staining (Biotium, USA). Results of real-time PCR employing SYBR Green (TTSuV1 and TTSuV2) were considered positive when C_t_ ≤ 37 and specific peaks with expected melting temperature of amplicons were observed.

Selected PCR products were sequenced by the Sanger method employing fluorescently labeled ddNTPs. The chromatograms were proofread by SeqMan Pro (DNASTAR Inc., USA). Sequences were aligned by ClustalW method and nucleotide and deduced amino acid identities were calculated using MegAlign software (DNASTAR Inc., USA). Program MEGA version 4 [24] employing Neighbor-joining method was used for the construction of phylogenetic trees.

Statistical analyses of data were performed by chi-square (χ^2^) tests with confidence limits of 95%, p < 0.05 using GraphPad Prism 5 for Windows (GraphPad Software, USA).

## Results

### Evaluation of virus prevalence in each animal group

Each of the five groups of pigs (diseased suckling, diseased post-weaning, diseased fattening, healthy fattening vaccinated and healthy fattening non-vaccinated pigs) was evaluated on the basis of the following parameters: 1) Individual detection of six viral pathogens (PCV2, PRRSV, TTSuV1, TTSuV2, PTV and PBoV1), 2) Prevalence of significant co-infections in each pig and each group and 3) Total number of viruses detected in each animal.

### Diseased suckling pigs

**Figure 1:**
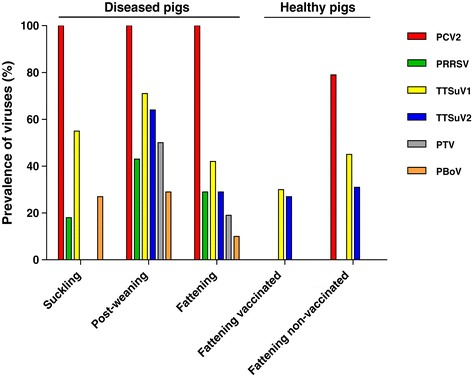
**Prevalence of viruses in individual groups of pigs.** Viruses were detected in pooled tissue samples (lymph nodes, liver and spleen) of diseased pigs of different age and lymph nodes of healthy animals. Total DNA or RNA was isolated by Chelex resins or TRIzol reagent, respectively. RNA was transcribed into cDNA using random hexamers (PTV) or gene-specific primer (PRRSV) and SuperScript III reverse transcriptase. Viral genomes were detected by PCR, RT-PCR or real-time PCR based on SYBR Green (TTSuV1 and TTSuV2).

**Figure 2:**
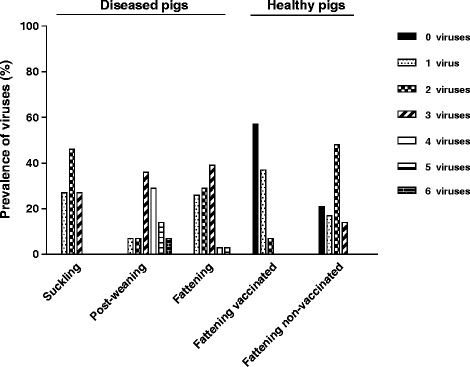
**Number of viruses detected in each group of pigs.** Number of viruses (PCV2, PRRSV, TTSuV1, TTSuV2, PTV, PBoV1) detected by PCR, RT-PCR or real-time PCR in tissue samples obtained from diseased and healthy animals were compared between different groups of pigs.

1. Beside PCV2 (100%), TTSuV1 was the most frequently detected virus (54.5%; Figure 1). PRRSV and PBoV1 infection was confirmed in 18.2% and 27.3% of suckling pigs, respectively. No animal was found to be positive for TTSuV2 or PTV.
2. In accordance with these findings, the most frequent co-infection was with PCV2 and TTSuV1 (54.5%; Table 2). The prevalence of co-infection with PCV2 and PRRSV reached 18.2%. Only one piglet was found to be simultaneously infected with PCV2, PRRSV and TTSuV1 and two piglets were co-infected with PCV2, PBoV1 and TTSuV1.
3. 45.5% of piglets were infected with two viruses (Figure 2) while in 27.3% of animals the presence of one or three viruses was confirmed. No piglet was found to be infected with more than three viral pathogens.

**Table 2:** **Percentage of selected co-infections in each group of pigs**

**Selected co-infections**	**Diseased pigs**			**Healthy pigs**	
	**Suckling**	**Post-weaning**	**Fattening**	Fattening*	**Fattening**
	**Less than 6 weeks**	**7-10 weeks**	**11-24 weeks**	**28 weeks**	**22 weeks**
	**n = 11**	**n = 14**	**n = 31**	**n = 30**	**n = 29**
PCV2 + PRRSV	18.2	42.9	29.0	0	0
PCV2 + TTSuV1	54.5	71.4	41.9	0	44.8
PCV2 + TTSuV2	0	64.3	29.0	0	31.0
PCV2 + TTSuV1 + TTSuV2	0	50	12.9	0	13.8
PCV2 + PRRSV + TTSuV1	9.1	28.6	6.5	0	0
PCV2 + PRRSV + TTSuV2	0	35.7	6.5	0	0
PCV2 + PRRSV + TTSuV1 + TTSuV2	0	21.4	0	0	0
PCV2 + PRRSV + PTV	0	28.6	0	0	0
PCV2 + PBoV1 + TTSuV1	18.2	28.6	6.5	0	0

*pigs vaccinated against PCV2 using Ingelvac CircoFlex vaccine (Boehringer Ingelheim, Germany).

### Diseased post-weaning pigs

1. All pigs were infected with PCV2 (100%), and they were also frequently infected with TTSuV1 (71.4%) or TTSuV2 (64.3%) (Figure 1). The prevalence of PRRSV, PTV and PBoV1 was also high (42.9%, 50.0%, and 28.6% respectively).
2. Most frequently, co-infections with PCV2 and TTSuV1 or TTSuV2 were found (71.4% or 64.3%, Table 2). Simultaneous detection of PCV2 and PRRSV (42.9%) was predominantly found in pigs suffering from the respiratory tract disorders. 83.3% of post-weanlings co-infected with PCV2 and PRRSV were found to be TTSuV2 positive as well. Among pigs simultaneously infected with PCV2 and PTV, almost 85.7% were also infected with TTSuV2, 71.4% with TTSuV1 and 57.1% with PRRSV. PBoV1 was detected only in pigs co-infected with PCV2 and TTSuV1.
3. 35.7%, 28.6% and 14.3% of post-weanlings were simultaneously infected with three, four and five viruses, respectively (Figure 2). One pig (7.1%) was affected with a single virus infection, i.e. PCV2 and one animal was infected with all six viruses.

### Diseased fattening pigs

1. The most prevalent virus detected was PCV2 (100%), followed by TTSuV1 that was detected in 41.9% of the pigs (Figure 1). PRRSV and TTSuV2 were both detected in 29.0% of the pigs, while 19.4% and 9.7% of pigs were infected with PTV and PBoV1, respectively (Figure 1).
2. Simultaneous infection of PCV2 with TTSuV1 was most frequently observed (41.9%; Table 2), followed by PCV2 with PRRSV and PCV2 with TTSuV2 co-infection at the same level (29.0%). The co-infections of PCV2, PBoV1 and TTSuV1 or PCV2, TTSuV1 and TTSuV2 were detected less frequently (Table 2).
3. Triple virus infection was observed in 38.7% of pigs, followed by double and single virus infection (29.0% and 25.8%, respectively; Figure 2). Simultaneous detection of more than three viruses was rare in this group of pigs.

### Healthy fattening vaccinated pigs

1. All healthy vaccinated animals were PCV2 free and no PRRSV, PTV and PBoV1 were detected. The prevalence of TTSuV1 and TTSuV2 in these animals reached the same value (30.0% and 26.7%, respectively) and was lower compared to the healthy non-vaccinated pigs (Figure 1).
2. 6.7% (2 out of 30) of these pigs were simultaneously infected with both TTSuVs.
3. Most pigs (56.7%) were not infected with any virus studied. Single virus infection was confirmed in 36.7% and two viral pathogens in a single pig was rare (6.7%; see Figure 2).

### Healthy fattening non-vaccinated pigs

1. 79.3% of animals infected with PCV2 did not show clinical signs of PMWS. The animals were not infected with PRRSV, PTV or PBoV1 but TTSuV1 and TTSuV2 were found in 44.8% and 31.0% of samples analyzed, respectively (Figure 1).
2. Co-infection of PCV2 with TTSuV1 was more frequently observed than co-infection of PCV2 with TTSuV2 (44.8% and 31.0%, respectively). Simultaneous detection of PCV2, TTSuV1 and TTSuV2 reached 13.8% (Table 2). TTSuV1 or TTSuV2 was not confirmed in PCV2-free pigs.
3. Infection with one, two and three viruses (17.2%, 48.3% and 13.8%, respectively) was observed (Figure 2). 20.7% of pigs were found to be negative for any virus tested.

### Comparison of virus prevalence between different groups of pigs

The results were further analyzed by comparing the different groups of diseased and healthy animals. Regarding the age, the post-weaning group of animals was found to be more infected with viruses than the other two age groups of diseased animals. Diseased pigs of all age groups were most frequently affected by triple virus infection (35.7%), but simultaneous co-infection of more than four viruses was not rare either (16.1%).

Prevalence of multiple infections was not significantly different between diseased fattening pigs (71.2% (23 out of 31)) and healthy fattening non-vaccinated pigs (62.1% (18 out of 29)); χ^2^ = 1.018; p = 0.3130). This observation was mostly due to similar co-infection of both groups of pigs with TTSuV1 and TTSuV2 and high infection with PCV2 (see Table 2). The comparison of healthy vaccinated fattening pigs with the same age category of diseased and healthy non-vaccinated pigs suggested strong benefit of vaccination against PCV2 (Figures 1 and 2). The observations between diseased fattening pigs and healthy vaccinated fattening pigs clearly showed a significant difference in prevalence of multiple viral infections (71.2% (23 out of 31) and 6.7% (2 out of 30), respectively; χ^2^ = 28.74; p < 0.0001). A significantly lower number of animals co-infected with two or more viruses was also found between healthy vaccinated pigs when compared to the healthy non-vaccinated animals (6.7% (2 out of 30) and 62.1% (18 out of 29), respectively; χ^2^ = 20.20; p < 0.0001).

### Genetic and phylogenetic analysis of PCR products

Since PCV2 and PRRSV circulating in Slovakia and the Czech Republic were genetically typed earlier [6,25,26], in the present study we focused on the newly identified viruses. Comparison of six TTSuV1 sequences from the untranslated region (UTR) revealed 86.6-96.3% nucleotide identity to each other. Five TTSuV2 sequences from the UTR were 96.1-99.0% identical to each other. Genetic and phylogenetic analysis of viral strains detected in Slovakia and the Czech Republic showed that the sequences clustered into TTSuVs lineages already identified by Cortey et al. [27] and Li et al. [28] (data not shown).

Two Slovak PTV sequences from the 5’-UTR were compared and a 99.4% similarity to each other was observed. Moreover, sequences were almost identical to the prototype strains of the PTV-7 serotype.

An alignment of two PBoV1 sequences of part of the VP1/VP2 genomic region obtained from isolates circulating in Slovakia confirmed a 99.8% identity at both the nucleotide and amino acid levels. Besides, nucleotide sequences were closely related to the first bocavirus isolated from pig sharing a 99.7-100% similarity.

## Discussion

The hypothesis that co-infection of PCV2 with other viral or bacterial pathogens might lead to PMWS development has been supported by several papers [9,13,29]. Our work was focused on the study of co-infections with six viral pathogens with the aim to extend information in this field. We realize that co-infection in pigs is much more complex and not limited to six viruses studied in this work. No doubt those animals could be infected with bacteria and other microorganisms which might influence investigated viral co-infections. Collection of samples obtained from dead animals suffering from PMWS at the terminal stage of disease gives at least partial information about the occurrence of six viruses in those pigs. Determination of particular viruses in pigs with or without PMWS revealed several interesting facts.

### PRRSV

The detection of PRRSV (PRRSV type 1, subtype EU-1) in PMWS pigs in Slovakia and Czech Republic [25,26] was not surprising because PRRSV is considered one of the major risk factors for a herd to be affected with PMWS [7]. Simultaneous occurrence of PCV2/PRRSV infection in pigs varies [11,30] and our observations on Slovak and Czech farms support these findings. The level of co-infection was the highest in post-weaning pigs and the lowest in suckling piglets. Detailed analysis revealed that all PRRSV-infected animals suffered from PRRSV/PCV2 co-infection.

Dual infection of pigs with PCV2 and PRRSV has consistently resulted in more severe disease than that caused by infection with either agent alone [29,31,32]. In the present study, it was observed that PCV2/PRRSV-infected pigs suffered from strong respiratory tract disorders on which an antibiotic treatment had no effect.

### TTSuVs

Although TTSuVs were frequently detected in PMWS pigs their role in the development of the diseases is not clear [33,34]. In general, contradictory results have been observed when co-infections of PCV2 and TTSuVs were studied. In some PMWS-affected pigs TTSuV2 was detected more frequently [14,35,36], while in others [33,37] and in the present study a higher prevalence of TTSuV1 was detected. In our healthy pigs limited to the fattening group (see Methods) the prevalence of TTSuV1 and TTSuV2 was similar. De Castro et al. [38] also did not find any significant relationship between TTSuVs and PCV2 in pigs in Brazil. The overall controversial results indicate that TTSuVs are unlikely to play an important role in the development of PMWS and our findings support this view.

It is not excluded that discrepancies in the results of different laboratories in the detection of TTSuVs in diseased and healthy pigs may also be related to the methods used for the detection of both viruses. Among molecular-genetic methods established for confirming TTSuVs in clinical samples, real-time PCR is often used because of its high detection sensitivity. Although a high prevalence of both TTSuVs was confirmed in our study also using real-time PCR, the lowest C_t_ values of positive samples were 30 (corresponding to 10^3^ copies of TTSuV1) and 23 (corresponding to 10^4^ copies of TTSuV2). Most positive samples had C_t_ values > 33, which represents 10^2^-10^1^of viral copies. It is really questionable if such amounts of virus can significantly contribute to the development of any disease, including PMWS.

### PTV

Porcine teschovirus is widely distributed in pig populations around the world [39]. Pigs from the present study, in which PTV was detected, most frequently suffered from diarrhea and inappetence with a loss of weight. Although porcine teschovirus may cause an asymptomatic disease, no healthy animal was found to be infected in our collection of samples. However, virus was frequently confirmed in clinically diseased post-weaning and fattening pigs.

In the last years, in almost all commercial pig herds PTV has been demonstrated in co-infections with other swine pathogens, most frequently PCV2 and PRRSV [40,41]. In the present study multiple infections of all three viruses was observed in 7.1% of diseased pigs only (4 out of 56).

### PBoV1

Since the discovery of a boca-like virus in PMWS-affected pig from Sweden [42], its presence has been confirmed in Europe [43], Asia [44] and Africa [45,46] with variable detection rates.

The role of PBoV1 alone in the development of the disease is still not clear, especially when the virus was confirmed in both apparently healthy and sick pigs. Moreover, the virus was detected along with PCV2 regardless of whether or not PMWS was diagnosed [16,47]. PCV2 and PBoV1 were simultaneously detected in 17.9% of our samples (10 out of 56) and only in pigs with clinical evidence of PMWS. Moreover, an interesting phenomenon was observed. In all PMWS-infected pigs in which PBoV1 alongside PCV2 was detected, TTSuV1 was also present. Of PMWS-suspected pigs co-infected with PBoV1, 60% were infected with PCV2 and TTSuV1. On the other hand, no healthy pig was positive for PBoV1. Simultaneous detection of PCV2, TTSuVs and PBoV1 in PMWS-affected pigs has been described earlier [16,47]. These findings indicate that PCV2 together with TTSuV1 might increase the susceptibility of pigs to bocavirus infection but this phenomenon has to be studied more deeply and with larger groups of pigs.

All single and multiple viral infections were related to the age of animals. Viruses were most frequently observed in pigs after weaning. On the contrary, suckling piglets had the lowest prevalence of each virus and multiple co-infections and this was the only group in which TTSuV2 was not detected. Very similar findings were described by de Castro et al. [38] in simultaneous infections caused by PCV2, TTSuV1 and TTSuV2. Thus, it is evident that maternal immunity due to colostrum feeding plays an important role in the protection of suckling pigs against viral infections.

The introduction of PCV2 vaccines has significantly changed the impact of PCV2 on global pig production [48–50]. In this study PCV2 was not found in tissues of healthy vaccinated pigs. This finding might be explained by combination of careful performance of vaccination using the effective vaccine and following of correct vaccination scheme. In addition, in slaughter-age pigs with a good health status their immune system is likely able to eliminate the virus. Since PCV2 detected in healthy non-vaccinated pigs reached 79.3% it is supposed that good management or the absence of some co-factor protected the healthy swine from a PMWS outbreak. Four of six viruses were not detected (PCV2, PRRSV, PTV and PBoV1) in these animals and more than 56% of pigs were not infected with any virus tested. By contrast, in the majority of healthy non-vaccinated pigs (62.1%; 18 out of 29) simultaneous detection of two and three viruses was confirmed. In addition, significantly lower number of animals co-infected with two and more viruses was found between vaccinated pigs compared to non-vaccinated ones (Figure 2). According to observations of Tshering et al., [51] TTSuVs seem not to be influenced by vaccination against PCV2. Lower number of TTSuV1-infected pigs was found in vaccinated animals when compared to the non-vaccinated ones, but the difference was not significant. The prevalence of TTSuV2 was almost the same between these groups of pigs.

Our study has shown that vaccination against PCV2 led to minimizing the risk of PMWS development and contributed to the decrease in susceptibility of pigs to the other viral pathogens.

What is the role of viruses such as TTSuVs, PBoV1, PTV or other newly discovered viruses in the development of significant porcine diseases with high economical impact? This question will become even more pertinent when novel pathogens are discovered by new molecular-genetic techniques, e.g. next generation sequencing. One may suggest that under certain conditions some viruses can cause significant disease, especially in an immunodeficient pig. Another possibility is based on the assumption that important pathogens, such as PRRSV or bacteria depress the immune system of swine and allow the entry and replication of other viruses. Another point of view is that certain pathogens may re-activate a persistent virus, thus, facilitating the development of a pathological process. Our findings that a significantly higher prevalence of all viruses has been found in diseased pigs confirm that multiple infections are associated with the presence of disease. Vaccination of pigs against PCV2 was also associated with lack of infection with other viruses.

The role of individual viral pathogens in multiple infections and their participation in the development of diseases has to be systematically studied. The first step, identification of multiple pathogens in diseased and healthy animals is just in progress. The next step requires carefully designed biological experiments with controlled infections with virus, bacteria and parasites in presence and absence of stress factors with the aim of finding combinations able to induce PMWS in pigs.

## Conclusions

The following results have been achieved in this study: i/post-weaning diseased pigs were more often co-infected with viral pathogens than suckling or fattening pigs, ii/clinical signs were stronger in PMWS pigs co-infected with PRRSV while the other viruses studied did not considerably influence the clinical manifestation of PMWS, iii/vaccination against PCV2 was associated with failure to detect PCV2 and significantly reduced viral co-infections in vaccinated pigs when compared to non-vaccinated pigs (diseased or healthy).

## Abbreviations

PCV2: Porcine circovirus type 2
PMWS: Post-weaning multisystemic wasting syndrome
PCVD: Porcine circovirus disease
PRRSV: Porcine reproductive and respiratory syndrome virus
PTV: Porcine teschovirus
TTSuV1: Torque teno sus virus type 1
TTSuV2: Torque teno sus virus type 2
PBoV1: Porcine bocavirus 1
PCR: Polymerase chain reaction
RT-PCR: Reverse transcription-polymerase chain reaction
UTR: Untranslated region
